# Appeasing Substance Administration at Feedlot Entry Impacted Temperament, Pen Behavior, Immunocompetence, and Meat Quality of Beef Heifers

**DOI:** 10.3390/ani14233517

**Published:** 2024-12-05

**Authors:** Douglas Gomes Vieira, Marcelo Vedovatto, Matheus Fellipe Ferreira, Juliana Ranches, Bruno Ieda Cappellozza, Osvaldo Alex de Sousa, Nelson Canuto, Marina de Nadai Bonin Gomes, Henrique Jorge Fernandes

**Affiliations:** 1 Faculdade de Medicina Veterinária e Zootecnia, Universidade Federal de Mato Grosso do Sul, Campo Grande 79070-900, MS, Brazil; douglas10dgv@gmail.com (D.G.V.); marina.bonin@ufms.br (M.d.N.B.G.); 2 Dean Lee Research and Extension Center, Louisiana State University, Alexandria, LA 71302, USA; 3 Hill Farm Research Station, Louisiana State University, Homer, LA 71040, USA; mferreira@agcenter.lsu.edu; 4 Eastern Oregon Agricultural Research Center, Oregon State University, Burns, OR 97720, USA; juliana.ranches@oregonstate.edu; 5 Novonesis, 2970 Hørsholm, Denmark; brie@novonesis.com; 6 Nutricorp, Araras 13601-000, SP, Brazil; desousaoa@gmail.com; 7 Unidade Universitária de Aquidauana, Universidade Estadual de Mato Grosso do Sul, Aquidauana 79200-000, MS, Brazil; nelsoncanuto@hotmail.com (N.C.); henrique.uems@hotmail.com (H.J.F.)

**Keywords:** beef heifers, ceruloplasmin, cortisol, immunity, meat quality, stress

## Abstract

This experiment evaluated the effects of bovine appeasing substance (BAS) administration at feedlot entry on growth, temperament, inflammation, response to vaccination, behavior, carcass characteristics, and meat quality of beef heifers. Thirty heifers were weaned and assigned to (d 0): (1) BAS (n = 15; SecureCattle; IRSEA Group) or (2) Saline (n = 15). On d 0, heifers were also vaccinated against respiratory diseases and slaughtered on d 150. Administering BAS increased (*p* = 0.05) average daily gain from d 6 to 45, reduced (*p* ≤ 0.03) plasma ceruloplasmin and serum cortisol concentrations on d 15 and 45 and increased (*p* = 0.03) the response to vaccination. Additionally, BAS reduced (*p* < 0.01) the entry scores on d 6, 15, and 45 and reduced (*p* = 0.05) exit scores on d 2, 6, and 15. The BAS increased (*p* ≤ 0.04) walking, drinking, and eating time, and tended (*p* ≤ 0.10) to increase lying and ruminating time. Lastly, BAS tended (*p* ≤ 0.10) to increase the myofibrillar fragmentation index and reduce the thiobarbituric acid reactive substance concentration in meat. Thus, BAS administration increased growth, reduced stress, and inflammation, and improved immune responses, behavior, and meat quality of heifers.

## Simple Summary

Beef cattle are exposed to several stressors during the first days in the feedlot, which impair their immunity, alter their behavior, and directly impact their carcass characteristics and meat quality. Strategies to mitigate stress upon arrival at the feedlot are needed, including the administration of bovine appeasing substance (BAS). The BAS is a mixture of fatty acids that replicate the composition of the original bovine appeasing pheromone and has been shown to alleviate the physiological consequences of stressful handling procedures in beef cattle. This experiment evaluated the effects of BAS administration at feedlot entry on growth, temperament, inflammation, response to vaccination, pen behavior, carcass characteristics, and meat quality of beef heifers. Our results show that BAS administration increased growth, reduced stress, and inflammation, and improved humoral immune responses, behavior, and meat quality of heifers.

## 1. Introduction

Weaning and feedlot entry are two of the most critical events in beef production systems, exposing animals to various stressors and health challenges [1]. These events stimulate adrenocortical and acute-phase protein responses that stimulate immediate and long-term impacts on calf growth, immunity, temperament, and meat quality [2,3]. In some scenarios, feedlot entry is conducted immediately after weaning and thus, strategies for mitigating different stressors in a short period are essential.

An alternative approach that can be used to alleviate specific stress-related responses is the use of appeasing pheromones. These have been studied and isolated in several species and act through chemoreceptors located in the vomeronasal organ and the olfactory epithelium, generating signals to the central nervous system to induce a behavioral and physiological response in a conspecific [4]. In cattle, a synthetic analog of bovine appeasing substance (BAS) has been created and is based on a mixture of fatty acids, reproducing the composition of the natural substance produced by the sebaceous gland on the skin of the mammary gland of the cow [4,5].

Recent research has described that BAS administration at weaning increased body weight (BW), reduced reactivity and serum cortisol concentration, and improved behavior and response to vaccination of grazing beef cattle [6]. In another experiment [3], BAS administration immediately before transporting the steers to the slaughterhouse reduced the meat pH and the risks of having dark, firm, and dry (DFD) cuts on those animals, which is often observed in high stressed animals, leading to reduced carcass and meat quality [7]. In a recent review conducted by Cappellozza et al. [5], the authors described that BAS administration has been evaluated in different commercial settings of beef and dairy cattle, with significant improvements for the performance and health of the herd, following an encounter with a stressful situation. Besides the results of these previous experiments, there is a lack of research evaluating the long-term effects of BAS administration at feedlot entry on growth, stress, temperament, carcass, and meat quality of beef heifers. Thus, we hypothesized that BAS administration at feedlot entry would reduce the stress and the stress-related effects on growth, physiological responses, temperament, pen behavior, and meat quality of beef heifers. Therefore, the objective of this experiment was to evaluate the effects of BAS administration at feedlot entry on growth, inflammation, response to vaccination, temperament, pen behavior, carcass characteristics, and meat quality of beef heifers.

## 2. Materials and Methods

### 2.1. Animals, Treatments, and Sample Collection

The experiment was conducted in an experimental feedlot located at Fazenda Agropecuária Cedron, Anastácio, MS, Brazil (20°30′03.5′′ S 55°53′34.1′′ W).

Thirty crossbreed weaned heifers [½ Nellore × ½ Angus; 198 ± 16.1 kg of BW; 7 ± 1 mo of age] were selected for the experiment. Those were previously raised on Marandu-grass pasture [Urochloa brizantha (Hochst. ex A. Rich) R. D. Webster, cv. Marandu] and on the day of weaning, were abruptly separated from their dams and transported 3 km in a single truck to the experimental feedlot. Afterward (d 0), heifers were stratified by BW and assigned to 1 of 2 treatments: (1) BAS (n = 15; SecureCattle; IRSEA Group, Quartier Salignan, France) or (2) Saline (n = 15; saline solution, 0.9% NaCl). The solutions were topically applied (5 mL/calf) to the nuchal skin area of each animal. Before the treatment administration, heifers were segregated into two groups (1 group/treatment). Saline-treated heifers were processed and immediately released to a feedlot pen before the BAS administration to the other group. This treatment administration method was chosen to avoid any cross-effects of BAS on Saline-treated heifers. Additionally, on d 0, before treatment administration, heifers were vaccinated against infectious bovine rhinotracheitis (IBR), parainfluenza-3 virus (PI3), bovine viral diarrhea virus type 1 (BVDV-1) and 2 (BVDV-2; 2 mL s.c.; Biopoligen HS; Biogenesis Bago SA, Buenos Aires, Argentina).

From d 0 to d 15, heifers were maintained in two similar pens (1 pen/treatment), separated from each other for about 200 m of distance to avoid cross-effects of BAS on Saline-treated heifers. On d 15, heifers were moved to a single pen (both treatments in the same pen), where they were kept until the end of the experiment (d 150).

Heifers were offered a total mixed ration (TMR) with three initial adaptation formulas to prevent metabolic disorders (1 week each; d 0 to 21) and one final ration (d 22 to 150; Table 1).

The TMR was provided ad libitum twice a day (07:00 a.m. and 02:00 p.m.), and the quantity offered was adjusted daily to ensure 5% residuals. The offered TMR samples were collected daily, frozen at −20 °C, and pooled by week for subsequent nutritional profile analysis.

Full BW and blood samples were collected on d 0, 2, 6, 15, 45, and 150. Fasted BW was not obtained to avoid shrink-induced stress effects on traits evaluated in this experiment [8]. Blood samples were collected from a jugular vein into two blood collection tubes (10 mL; Vacutainer, Becton Dickinson, Franklin Lakes, NJ, USA) with and without sodium heparin for collection of plasma and serum, respectively. After collection, blood samples were immediately stored on ice and then centrifuged at 1200× *g* for 30 min for plasma and serum harvest. Samples were stored at −20 °C until further analysis of plasma concentrations of haptoglobin and ceruloplasmin and serum concentration of cortisol and antibody titters (i.e., against IBR, PI3, and BVDV-1 and 2). Haptoglobin, ceruloplasmin, and cortisol were analyzed from samples collected on d 0, 2, 6, 15, 45, and 150, while antibody titers were analyzed in samples from d 0, 15, and 45.

Three trained technicians evaluated the temperament (i.e., the same trained personnel blinded to treatments during the entire experiment) in the corral on d 0, 2, 6, 15, 45, and 150. The entry and exit scores in the squeeze chute were evaluated according to Baszczak et al. [9], with scores 1 = animals that walked in or out of the chute; 2 = those that trotted to or from the chute; and 3 = those that ran or galloped in or out of the chute. The chute score was evaluated using an adaptation of Cooke et al. [10] criterion, where 1 = calm with no movement; 2 = restless movements; 3 = frequent movement; 4 = constant movement, vocalization, shaking of the chute; and 5 = violent and continuous struggling. All heifers were handled calmly and smoothly without an electric shock or physical contact until they reached or left the chute.

On d 0, heifers were individually identified on both sides of the body, with large numbers using hair dye to facilitate animal identification for behavioral evaluation. Behavior was evaluated for nine consecutive days (d 0 to d 9) during twelve consecutive hours [from 0600 h to 1800 h; except for the days of handling in the corral (d 0, 2, and 6), when heifers were evaluated only after the handling (i.e., from 1000 h to 1800 h)] with an interval of 5 min between each scan. The variables evaluated were adapted from Enríquez et al. [11]: walking, drinking water, eating, lying, lying ruminating, standing idle, standing ruminating, playing (jumping, running, no sign of stress), seeking (walking beside the fence, with head held high, looking for the dam), and vocalizing. The variables of total lying (lying + lying ruminating), total standing (standing idle + standing ruminating), and total ruminating (lying ruminating + standing ruminating) were later calculated.

On d 150, all heifers were loaded into a single truck and transported for 115 km to a commercial slaughterhouse. Heifers were slaughtered using the technique of brain concussion and section of the jugular vein. After slaughter, the carcass of each heifer was divided into two halves, weighed, and refrigerated (2 to 4 °C) for 24 h. After this period, pH and temperature were collected in the semimembranosus muscle using a pH meter (Mettler M1120x; Mettler Toledo, Columbus, OH, USA) and thermometer (Clink Termômetro Digital; Clink Comércio de Importação e Exportação LTDA; Joinvile, SC, Brazil) devices. Then, a transverse cut was performed in the longissimus dorsi muscle between the 12th and 13th ribs to assess the subcutaneous fat thickness and ribeye area (REA). The subcutaneous fat thickness was measured using a digital caliper (Stainless Steel Caliper; Dexter, China). The REA was determined by taking its format on a tracing paper with the area evaluated in an area meter equipment (LI-3100 Area Meter, LI-COR Environmental, Lincoln, NE, USA).

Ribeye samples (2.5 cm thick) were collected from the longissimus dorsi muscle (between the 12th and 13th ribs), vacuum-packaged, identified, and frozen at −20 °C for subsequent determination of the Warner–Bratzler shear force (WBSF), marbling score, fat color, meat color, exudate loss, cooking loss, myofibrillar fragmentation index (MFI), and thiobarbituric acid reactive substances (TBARS) concentration.

### 2.2. Laboratory Analysis

The TMR samples were dried for 72 h at 55 °C and ground through a 1 mm sieve AOAC [12]. Then, these were analyzed according to AOAC [12] for crude protein (method 976.05), ash (method 942.05), and ether extract (method 920.39). The concentrations of neutral and acid detergent fiber were analyzed as described by Van Soest et al. [13]. The total digestible nutrient concentrations were calculated as described by Weiss et al. [14], and net energy for maintenance and gain by the equations proposed by the NASEM [15]. The nutritional profile of TMR diets is described in Table 1.

Plasma concentrations of haptoglobin were analyzed as described by Cooke and Arthington [16] and ceruloplasmin as described by Demetriou et al. [17]. The inter- and intra-assay CV were 3.6% and 5.4% for haptoglobin and 2.2% and 4.7% for ceruloplasmin, respectively. The serum concentration of cortisol was analyzed (Immulite 1000; Siemens Medical Solutions Diagnostics, Los Angeles, CA, USA) as previously described by Cooke et al. [18] due to 100% cross-reactivity between bovine and human cortisol and accomplished within a single assay with an intra-assay with a CV of 7.6%.

Antibody titers against IBR, PI3, and BVDV-1 and 2 viruses were assessed using procedures outlined by Rosenbaum et al. [19]. Individual serum samples were evaluated for the greatest dilution of antibody titers that achieved total protection of cells against those viruses and are reported as log2. Heifers with antibody titers ≥ 4 for each virus were considered seropositive and assigned a value of 1, whereas those with antibody titers < 4 were considered seronegative and assigned a value of 0. These scores were utilized to determine the percentage of heifers that had positive seroconversion for antibody protection against the aforementioned viruses, as previously described by Richeson et al. [20].

Meat samples were evaluated for tenderness through the WBSF method AMSA [21] using six replicates (1.27 cm diameter), with the fiber direction parallel to the longest dimension of the strip and perpendicular to the direction of the blade, using WBSF equipment (G-R Manufacturing Co., Manhattan, KS, USA) equipped with a Warner–Bratzler blade. Marbling was scored according to the USDA Quality Grade in six classes, where slight = 400, small = 500, modest = 600, moderate = 700, slightly abundant = 800, and moderately abundant = 900. Fat and meat color were evaluated using a colorimeter (MiniScan XE Plus; HunterLab, Reston, VA, USA) with a D65 light source, with 10° of observation angle and 30 mm opening of the measuring cell. The luminosity (L*), redness (a*), and yellowness (b*) scales of the CIE Lab system were used.

Meat samples were evaluated for exudate and cooking losses following the methodologies reported in AMSA [21]. Briefly, each steak was weighed on aluminum trays and thawed for 24 h at 4 °C to obtain the exudate losses. The steaks were then cooked in an oven with upper and lower heaters (Forno Elétrico Crystal Plus Advanced, Layr Eletrodomésticos, São Paulo, SP, Brazil) at 170 °C until reaching 71 °C in the center. The temperature was determined with individual thermocouples thermometers (Taylor 1478-21, Taylor Precision Products, Oak Brook, IL, USA) inserted into the geometric center of each pile. The samples were removed from the oven, dried, and weighed to obtain the cooking losses. The MFI was evaluated using the procedures described by Ramos [22] and TBARS concentration according to Lemon [23].

### 2.3. Statistical Analyses

One Saline-treated heifer had to be removed from the experiment because of its temperament, which challenged her handling in the working chute. The heifers were considered the experimental units for all analyses. Normal distribution and homogeneity of variance were analyzed using the UNIVARIATE procedure with the NORMAL option in SAS (version 9.4; SAS Inst. Inc., Cary, NC, USA), and all data were normally distributed (*p* ≥ 0.33; Shapiro–Wilk test). Data were analyzed using the MIXED (for quantitative variables) and GLIMMIX (for binomial variables) SAS procedures. The Satterthwaite approximation was selected to determine the denominator of degrees of freedom for the test of fixed effects. Average daily gain (ADG), carcass traits, and meat quality traits were tested using the following statistical model:

*Y_ij_* = *μ* + *T_i_* + *C_j_*(*T_i_*) + *ϵ_ij_*,

where *Y_ij_* represents the response variable for calf *_j_* in treatment *_i_*, *μ* is the overall mean, *T_i_* is the fixed effect of treatment (fixed), *C_j_*(*T_i_*) is the effect of calf nested within treatment (random), and *ϵ_ij_,* is the residual error.

All other variables were analyzed as repeated measures and were tested using the following statistical model:

*Y_ijk_* = *μ* + *T_i_* + *C_j_*(*T_i_*) + *D_k_* + (*T* × *D*)*_ik_* + *βX_ijk_* + *ϵ_ijk_*,

where *Y_ijk_* represents the response variable for calf *_j_* in treatment *_i_*, of day *_k_*, *μ* is the overall mean, *T_i_* is the fixed effect of treatment (fixed), *C_j_*(*T_i_*) is the effect of calf nested within treatment (random), *D_k_* is the fixed effect of day (fixed), (*T × D*)*_ik_* is the interaction between treatments and days (fixed), *βX_ijk_* is the covariate (the results of d 0 were included as covariates in each respective analysis but were removed from the model when *p* > 0.10; fixed) and *ϵ_ijk_* are the residual errors. The Toeplitz covariance structure was selected for BW, and the first-order autoregressive covariance structure was selected for all other variables. The covariance structures were selected according to the lowest Akaike information criterion. Means were separated using the protected least significant difference (PDIFF; *t*-test), and all results were reported as least squares mean (LSMEANS) followed by the standard error of the mean (SEM). Significance was defined as *p* ≤ 0.05, and tendency when *p* > 0.05 and ≤0.10.

## 3. Results

No treatment × day interaction (*p* = 0.29) or main treatment effects (*p* = 0.88) were detected for BW. However, BAS-treated heifers had greater ADG (treatment effects; *p* = 0.05) from d 6 to 45 than Saline-treated heifers (Table 2).

**Figure 1:**
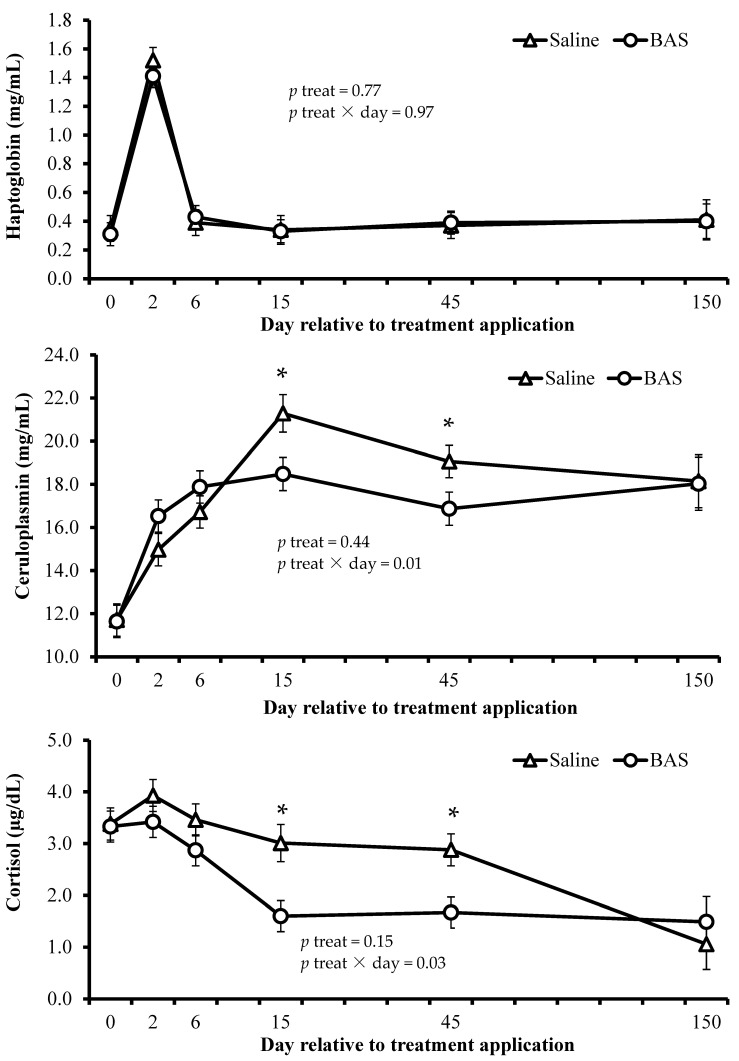
Plasma concentrations of haptoglobin and ceruloplasmin and serum concentration of cortisol of beef heifers receiving saline solution (Saline; n = 14) or bovine appeasing substance (BAS; n = 15). * *p* ≤ 0.05.

However, BAS-treated heifers had lower plasma concentrations of ceruloplasmin and serum concentrations of cortisol (treatment × day effects; *p* ≤ 0.03) on d 15 and 45, compared to Saline-treated heifers (Figure 1). Furthermore, BAS-treated heifers had greater IBR titer concentration (treatment × day effects; *p* = 0.03) on d 15 and 45 and tended to have greater IBR seroconversion (treatment × day effects; *p* = 0.09) on d 15 vs. Saline-treated heifers (Table 3).

In addition, BAS-treated heifers tended to have greater PI3 titer concentration (treatment × day effects; *p* = 0.09) on d 45, whereas no effects were noted (*p* ≥ 0.21) for PI3 seroconversion (Table 3). Surprisingly, despite being present in the vaccine, titer against BVDV-1 and BVDV-2 was not produced by any heifer, regardless of the treatment.

The BAS-treated heifers had lower entry scores on d 6, 15, and 45 (treatment × day effects; *p* < 0.01), tended to have lower chute scores from d 2 to 150 (treatment × day effects; *p* = 0.08), and had lower exit scores on d 2, 6, and 15 (treatment × day effects; *p* = 0.05) compared to Saline-treated heifers (Table 4).

The diurnal behavior was affected by treatments and when evaluated in min/d, BAS-treated heifer spent more time walking, drinking, and eating (treatment effects; *p* ≤ 0.04), tended to have greater total time lying and ruminating (treatment effects; *p* ≤ 0.10), while spending less time standing idle, total standing and seeking (treatment effects; *p* < 0.01). Finally, BAS-treated heifers tended to spend less time vocalizing (treatment effects; *p* = 0.10; Table 5).

Treatments did not affect carcass characteristics (*p* ≥ 0.11), neither WBSF, marbling score, fat color, meat color, exudate, or cooking losses (Table 6). However, BAS-treated heifers tended (*p* ≤ 0.10) to have greater MFI and lower TBARS concentration vs. Saline-treated heifers (Table 6).

## 4. Discussion

### 4.1. BAS Effects on Growth

The elevated ADG during the first two weeks of the experiment for both treatments is likely due to gastrointestinal tract refilling. Previous stress from weaning and transportation likely reduced feed intake, leading to gastrointestinal tract emptying, which was reversed as the animals started consuming the feedlot diet. The BAS administration improved the ADG from d 6 to 45 but did not improve the BW at slaughter (d 150). Several experiments reported an improvement in ADG in the weeks post-BAS administration (~4 to 6 wk; [3,6,24,25]) but not for a longer time (100 d; [6]). In this experiment, the lack of observable BAS effects after day 45 is likely attributed to the product duration of action, which the manufacturer states last approximately 15 days. The enhanced ADG observed in heifers treated with BAS may result from a reduced stress response, as evidenced by lower temperament scores, decreased serum cortisol concentration, and reduced plasma ceruloplasmin concentrations. These changes likely influenced behavior by increasing the time spent eating and ruminating while reducing activities such as seeking and vocalizing, suggesting a quicker adaptation to the new environmental conditions.

### 4.2. BAS Effects on Acute-Phase Proteins and Cortisol

Administering BAS reduced the plasma concentrations of ceruloplasmin and serum cortisol concentrations on d 15 and 45. In other experiments, BAS administration also reduced cortisol serum, plasma, or hair concentrations [6,25,26], but not on plasma ceruloplasmin [6]. Our experiment demonstrated that weaning and feedlot entry elicited an adrenocortical response (i.e., increasing the serum concentration of cortisol) and were responsible for eliciting an inflammatory response (i.e., increasing the plasma concentration of ceruloplasmin and haptoglobin), suggesting that BAS was effective in attenuating this cascade of events. According to Cappellozza et al. [5], acute increases in cortisol have been reported to trigger an acute, transient, and temporary inflammatory cascade, and it has been associated with reduced growth rates.

The exact mechanism by which BAS administration reduces cortisol production remains unclear. However, it is understood that BAS interacts with organs involved in pheromone detection, such as the main olfactory epithelium (MOE) and the vomeronasal organ (VNO) [5,27]. The MOE recognizes general odor molecules and non-specific environmental signals, while the VNO specializes in detecting pheromones, transmitting specific chemical signals via receptors [28]. This process triggers a neuroendocrine cascade [5]. Neurons in the VNO can encode the intensity of a stimulus, activating a specific neural subpopulation and transmitting an electrochemical signal to the brain [27]. This signal stimulates the hypothalamus to produce a neuroendocrine response tailored to the neural subpopulation activated, leading to calming effects in the animal [5].

### 4.3. BAS Effects on Response to Vaccination

The increase in IBR and PI3 titer concentrations following BAS administration suggests an improvement in the immune system by the product. In another experiment where the stress amount likely was alleviated (i.e., animals were kept in the same location and grazing the same pasture before and after weaning), BAS administration at weaning concurrently with vaccination against respiratory diseases increased the serum titer concentration against PI3 and BVDV-1 [6]. The improvement of the immune system in BAS-treated heifers is related to less adrenocortical stimulation (i.e., less production of cortisol). Cortisol affects the immune system in several ways, including reducing immune cell proliferation and differentiation, affecting cell function, and increasing cytokine expression [2].

### 4.4. BAS Effects on Temperament and Pen Behavior

Heifers that received BAS presented an alleviated post-weaning stress response and lower temperament scores (mainly chute scores) for 150 d. However, the most intense effects were observed for the first 45 d of the experiment. Over 100 days, Vieira et al. [6] observed a reduced temperament score for only 51 d in calves that received a BAS administration at weaning. The current experiment likely had more stressful weaning practices than the ones reported by Vieira et al. [6] (i.e., including transportation and change from grazing to a feedlot environment), and this greater stress could have caused emotional trauma, and, thus, becoming more reactive for a longer period. This could explain the lower production of cortisol and ceruloplasmin, leading to a decrease in temperament score observed in BAS-treated heifers.

The BAS administration influenced pen behavior by increasing the time heifers spent walking, drinking, eating, lying, and ruminating, while reducing the time spent standing, seeking, and vocalizing. Similar behavioral effects were observed by Schubach et al. [26], where BAS-treated calves at weaning, housed in a feedlot, tended to engage in more allogrooming and exhibited increased step activity post-weaning. Likewise, in the study by Vieira et al. [6], BAS-treated calves weaned into a grazing system spent more time grazing, eating concentrate, walking, and standing ruminating, while spending less time lying down, consistent with findings from the current experiment. Collectively, these results suggest that BAS-treated heifers were more active following weaning. The increased walking observed in this study appeared to reflect exploratory activity rather than heightened stress. Additionally, the greater time spent drinking and eating in BAS-treated heifers indicates a quicker adaptation to the new environment, likely due to reduced stress levels.

The BAS-treated heifers presented less time seeking and vocalizing. An increase in time seeking for the dam and vocalizing are traditional characteristics of behavior presented by post-weaning calves due to the high psychological stress caused by the separation from their dams [11]. In other experiments, BAS administration at weaning reduced the number of escape attempts and mounts on subsequent days after weaning [26] and increased the time playing and decreased the time vocalizing [6]. All these effects on behavior observed herein could be explained by the reduced temperament, serum cortisol, and plasma ceruloplasmin concentrations in BAS-treated heifers.

### 4.5. BAS Effects on Carcass and Meat Quality

Stressful conditions are frequently observed during the process of transporting animals to slaughter. This is due to the various management procedures involved, including human handling, transportation to the slaughter facility, loading and unloading, exposure to an unfamiliar environment, and periods of feed and water deprivation [3]. Furthermore, more stressed animals before slaughter had reduced carcass and meat quality [7]. However, in the current experiment, BAS administration did not affect carcass characteristics, WBSF, marbling score, fat color, meat color, exudate loss, or cooking loss. In an experiment conducted by Cappellozza et al. [3], BAS administration immediately before transporting steers to the slaughterhouse reduced the meat pH and the risks of having DFD meat but did not affect the meat color. In our experiment, the lack of effects on several variables of carcass and meat quality could be related to a very long period of BAS administration before the slaughter (150 d), reducing the chance to detect effects, compared to Cappellozza et al. [3] that applied this technology immediately before transportation to the slaughterhouse.

In this experiment, BAS administration tended to increase MFI in meat. The MFI is a key indicator of enhanced meat tenderness and proteolysis, reflecting the breakdown of the I-band and the loss of myofibril integrity [29], and stress normally reduces the MFI by affecting the pH and glycogen concentration in the muscle [30]. In the current experiment, BAS administration did not reduce the serum concentration of cortisol on the day before the slaughter (d 150) but tended to decrease chute scores during the entire experiment, including d 150. As BAS-treated heifers were calmer during the entire experiment, we hypothesize that they were also calmer in the slaughterhouse, thus storing less glycogen in the muscle and improving the MFI by this mechanism.

The BAS administration tended to reduce TBARS concentration in meat. The TBARS concentration in meat measures lipid oxidation, and it is a primary cause of quality defects in meat products, including changes in flavor, color, texture, and nutritive value [31]. According to the same authors, transit and handling in the slaughter facilities increase oxidative stress through psychological stress, feed and water deprivation, and physical exertion. As BAS-treated heifers were calmer during the entire experiment, we hypothesize they were also calmer during transportation and slaughter facilities and this stress reduction was responsible for reducing the TBARS concentration in meat. We did not evaluate temperament or collected blood samples (i.e., to evaluate cortisol concentration and oxidant/antioxidant system markers) during transportation and in the slaughterhouse to test this rationale, and it deserves further investigation. Research is still warranted to examine the benefits of further repeated BAS administration (i.e., at weaning and before transportation to the slaughter facilities on temperament, physiological stress, oxidative stress, and meat quality of beef cattle.

## 5. Conclusions

In summary, BAS administration reduced the impact of weaning and feedlot entry stress on growth (d 6 to 45), immune system, serum cortisol concentration, plasma ceruloplasmin concentration, improved the behavior and adaptation to a new environment (i.e., feedlot pen), and no effect on the carcass and improved meat quality (i.e., increased MFI and decreased TBARS concentration on meat). This experiment shows the potential of BAS to alleviate stress and its negative impact on the performance, welfare, and meat quality of confined beef heifers.

## Figures and Tables

**Table 1:** Composition and nutritional profile of the total mixed ration offered ad libitum to the heifers.

Items	Periods			
	d 0 to 7	d 8 to 14	d 15 to 21	d 22 to 150
Composition, dry matter (DM) basis				
Grass silage ^1^, %	80.7	66.3	43.1	12.0
Sugar cane bagasse, %	0.00	0.00	0.00	7.16
Ground corn, %	10.0	24.9	37.7	59.7
Soybean hull, %	6.00	4.14	13.5	14.3
Soybean meal, %	1.60	2.48	2.70	2.60
Commercial mix ^2^, %	1.70	2.24	3.00	4.29
Nutritional profile ^3^, (DM) basis				
CP, %	9.61	10.8	12.5	14.0
Ash, %	7.89	6.93	5.81	4.10
EE, %	2.27	3.02	3.74	4.86
NDF %	63.4	53.5	45.9	32.5
ADF, %	39.9	33.0	28.1	19.3
TDN ^4^, %	53.9	59.0	66.1	73.9
Nem ^5^, Mcal/kg	1.37	1.45	1.54	1.63
Neg ^5^, Mcal/kg	0.79	0.86	0.96	1.04

^1^ Panicum maximum cv. Tanzânia; ^2^ Nutriforte Concentrado Confinamento (Nutriforte, Dourados, MS. Brazil). Composition: crude protein (CP) = 90%; total digestible nutrients (TDN) = 67%; ether extract (EE) = 0.7; calcium = 0.25%; phosphorus = 0.14%; sodium = 0.25%, potassium = 8500 mg/kg; cobalt = 1.0 mg/kg; selenium = 2.0 mg/kg; zinc = 450 mg/kg; and sodium monensin = 600 mg/kg; ^3^ neutral detergent fiber (NDF); acid detergent fiber (ADF), net energy for maintenance (NEm) and gain (NEg); ^4^ calculated as proposed by Weiss et al. (1992); ^5^ calculated using the equations proposed by the NASEM (2016).

**Table 2:** Growth performance of beef heifers receiving saline solution (Saline; n = 14) or bovine appeasing substance (BAS; n = 15).

Items	Treatments		SEM	*p*-Value	
	Saline	BAS		Treatment	Treatment × Day
Body weight, kg				0.88	0.29
d 0	198	198	3.67		
d 6	214	211	3.67		
d 15	231	232	3.67		
d 45	275	280	3.72		
d 150	420	418	3.67		
Average daily gain, kg/d					
d 0 to 6	2.62	2.19	0.27	0.27	
d 0 to 15	2.18	2.29	0.14	0.58	
d 0 to 45	1.70	1.84	0.09	0.27	
d 0 to 150	1.48	1.47	0.04	0.82	
d 6 to 15	1.88	2.35	0.16	0.05	
d 6 to 45	1.56	1.79	0.08	0.05	
d 6 to 150	1.43	1.44	0.04	0.93	
d 15 to 45	1.46	1.63	0.10	0.26	
d 15 to 150	1.40	1.37	0.04	0.61	
d 45 to 150	1.39	1.32	0.04	0.31	

No treatment × day interaction or main treatment effects were detected (*p* ≥ 0.77) for plasma concentration of haptoglobin (Figure 1).

**Table 3:** Response to vaccination of beef heifers receiving saline solution (Saline; n = 14) or bovine appeasing substance (BAS; n = 15).

Items ^1^	Treatments		SEM	*p*-Value	
	Saline	BAS		Treatment	Treatment × Day
**IBR**					
Titers, log_2_				<0.01	0.03
d 0	0.00	0.00	0.35		
d 15	1.50 ^a^	3.17 ^b^	0.35		
d 45	2.17 ^a^	3.84 ^b^	0.35		
Seroconversion, % total				0.12	0.09
d 0	0.00	0.00	8.60		
d 15	66.7 ^x^	100 ^y^	8.60		
d 45	100	100	8.60		
PI3					
Titers, log_2_				0.10	0.09
d 0	0.32	0.57	0.41		
d 15	3.59	4.50	0.41		
d 45	2.87 ^x^	4.67 ^y^	0.41		
Seroconversion, % total	75.0	100	13.0	0.21	0.34

^1^ On d 0, before treatment administration, heifers were vaccinated against infectious bovine rhinotracheitis (IBR), parainfluenza-3 (PI3) virus, bovine viral diarrhea virus type 1 and 2 (BVDV-1 and 2; 2 mL s.c.; Biopoligen HS; Biogenesis Bago SA, Buenos Aires, Argentina); ^x–y^ Within a row, without a common superscript tends to differ (*p* ≤ 0.10). ^a,b^ Within a row, without a common superscript differ (*p* ≤ 0.05).

**Table 4:** Temperament scores in the chute of beef heifers receiving saline solution (Saline; n = 14) or bovine appeasing substance (BAS; n = 15).

Items	Treatments		SEM	*p*-Value	
	Saline	BAS		Treatment	Treatment × Day
Entry Score, 1–3				0.02	<0.01
d 0	1.34	1.40	0.07		
d 2	1.16	1.08	0.07		
d 6	1.46 ^a^	1.17 ^b^	0.07		
d 15	1.50 ^a^	1.17 ^b^	0.07		
d 45	1.63 ^a^	1.31 ^b^	0.07		
d 150	1.30	1.45	0.07		
Chute score, 1–5				0.01	0.08
d 0	1.71	1.69	0.16		
d 2	2.10 ^x^	1.69 ^y^	0.16		
d 6	2.12 ^x^	1.47 ^y^	0.16		
d 15	1.97 ^x^	1.46 ^y^	0.16		
d 45	1.85 ^x^	1.45 ^y^	0.16		
d 150	1.86 ^x^	1.47 ^y^	0.16		
Exit score, 1–3				0.01	0.05
d 0	1.69	1.60	0.11		
d 2	1.63 ^a^	1.30 ^b^	0.11		
d 6	1.79 ^a^	1.40 ^b^	0.11		
d 15	1.76 ^a^	1.25 ^b^	0.11		
d 45	1.77	1.61	0.11		
d 150	1.62	1.55	0.11		

^a–b^ Within a row, without a common superscript differ (*p* ≤ 0.05); ^x–y^ Within a row, without a common superscript tends to differ (*p* ≤ 0.10).

**Table 5:** Pen behavior of beef heifers receiving saline solution (Saline; n = 14) or bovine appeasing substance (BAS; n = 15).

Items	Treatments		SEM	*p*-Value
	Saline	BAS		
min/day				
Walking	37.8	53.1	2.34	<0.01
Drinking water	9.52	11.9	0.81	0.04
Eating	126	145	4.09	<0.01
Lying	115	119	4.34	0.50
Lying ruminating	57.1	67.9	4.73	0.11
Total lying	172	187	6.45	0.10
Standing idle	207	170	5.74	<0.01
Standing ruminating	15.6	14.9	2.22	0.82
Total standing	223	185	7.25	<0.01
Total ruminating	72.8	82.8	4.11	0.09
Playing	5.91	4.81	0.92	0.40
Seeking	4.64	1.30	0.32	<0.01
Vocalizing	9.56	5.96	1.54	0.10
% of the activities				
Walking	6.61	11.1	0.45	<0.01
Drinking water	1.55	1.95	0.13	0.03
Eating	20.5	23.3	0.69	<0.01
Lying	19.4	19.8	0.81	0.69
Lying ruminating	9.13	10.5	0.74	0.20
Total lying	28.6	30.3	1.13	0.26
Standing idle	36.5	28.7	1.17	<0.01
Standing ruminating	2.43	2.28	0.35	0.76
Total standing	38.9	31.0	1.39	<0.01
Total ruminating	11.6	12.8	0.65	0.20
Playing	0.94	0.75	0.14	0.36
Seeking	0.93	0.23	0.06	<0.01
Vocalizing	2.05	1.32	0.45	0.26

When the behavior was evaluated in percentual of the activities, BAS-treated heifers had greater percentual walking, drinking, and eating (treatment effects; *p* ≤ 0.03) and lower percentual standing idle and seeking (treatment effects; *p* < 0.01; Table 5). Treatments did not affect other behavior variables in min/d or percentual of activities (*p* ≥ 0.11; Table 5).

**Table 6:** Carcass characteristics and meat quality of beef heifers receiving saline solution (Saline; n = 14) or bovine appeasing substance (BAS; n = 15).

Items ^1^	Treatments		SEM	*p*-Value
	Saline	BAS		
Carcass traits				
Hot carcass weight, kg	220.54	221.33	3.45	0.87
Carcass yield, %	51.75	51.92	0.27	0.67
pH (24 h)	5.77	5.76	0.06	0.94
Temperature (24 h), °C	7.60	8.02	0.19	0.11
Fat thickness, mm	6.99	7.99	0.45	0.13
Ribeye area, cm^2^	71.9	72.8	1.91	0.76
Meat quality traits				
WBSF, kgF/cm^2^	7.20	6.91	0.47	0.68
Marbling score, points	430.0	440	15.8	0.66
Fat color				
L*	65.0	65.4	0.51	0.57
a*	6.57	6.95	0.27	0.34
b*	11.4	11.5	0.33	0.84
*Meat color*				
L*	39.9	39.3	0.84	0.60
a*	18.4	18.1	0.66	0.77
b*	8.95	8.33	0.52	0.41
Exudate loss,%	4.90	4.83	0.51	0.93
Cooking loss,%	21.7	25.6	1.67	0.12
MFI	89.2	97.9	3.54	0.10
TBARS, mg/g	9.19	9.09	0.03	0.06

^1^ Warner–Bratzler shear force (WBSF), luminosity (L*), green to red (a*), and blue to yellow (b*), thiobarbituric acid reactive substances (TBARS); myofibrillar fragmentation index (MFI).

## Data Availability

The original contributions presented in this study are included in the article. Further inquiries can be directed to the corresponding author.
